# Development of research integrity in France is on the rise: the introduction of research integrity officers was a progress

**DOI:** 10.1186/s41073-019-0080-8

**Published:** 2019-10-16

**Authors:** Hervé Maisonneuve

**Affiliations:** 1 Scientific Committee, IRAFPA (Institute of Research and Action on Fraud and Plagiarism in Academia), Geneva, Switzerland; 2 H2MW, 30 rue Faidherbe, 75011 Paris, France

**Keywords:** Research integrity, Research integrity officers

## Abstract

**Background:**

Implementing responsible conduct of research and monitoring bad practices requires time and tact. In France, it was in 2015 that the wishes of those in charge of research proposed the appointment of research integrity officers (RIOs) in all universities, national higher education schools, and research institutions. Our objectives were to search for information to describe the RI development and to analyze the RIOs’ profiles.

**Methods:**

The OFIS (Office Français de l’Intégrité Scientifique) website lists all public research institutions and universities (RIUs) and their designated RIOs. During the period between 6 and 14 October 2018, and on 2 May 2019 (updating), we entered two keywords (“plagiat” and “intégrité”) into the search engines on the RIU homepages and retrieved the relevant information (including “research” and “scientific” integrity when “intégrité” was entered). We consulted the governance and downloaded the organigram to determine whether the RIO positions and names were mentioned. We searched for the domains of expertise, sex, and age of the RIOs from their CVs (institutional websites), LinkedIn profiles, and various Google links.

**Results:**

The OFIS website lists 142 RIUs. Searching for the keyword “plagiarism” retrieved 25 RIUs; however, the web information was minimal, and consisted entirely of charters, interviews, and rare training modules. The keyword “integrity” turned up 23 RIUs. Nonetheless, there was little information available beyond notices for seminars, events, and a few training modules. Of the total 142 RIUs, 66.2% (*n* = 94) had named 96 RIOs. Furthermore, 29.2% RIOs (*n* = 28) were female and 70.8% (*n* = 68) were male; 38 RIOs were retired (> 65 years old) 58 were active (< 65 years old), and had a RIO function added to their usual laboratory activities.

**Conclusion:**

There is a lack of information about RI on the websites of French universities and research organizations, which may reflect a lack of information and commitment in the institutions themselves.

## Introduction

National policies to avoid questionable research practices (QRPs) and responsibly conduct research were lacking in the French scene in 2014. The two main national research bodies included a few organizations like the CNRS (Centre National de la Recherche Scientifique), which installed the Comets (Ethical committee) in 1994 with no power to investigate fraud or QRPs; and the Inserm (Institut national de la santé et de la recherche médicale), which created the “Délégation à l’intégrité scientifique” in 1999 with the power to investigate misconduct within the institution. Inserm and CNRS are two leading research institutions in France. They were the driving force behind the promotion of the national charter, together with university representatives. It is a partnership to develop research integrity, without a position of authority between institutions.

On 16 January, 2015, a national charter on the ethics within the research profession was issued and signed by leading public French research institutions and universities (RIUs) [1] Each public research and higher educational institution should implement clear procedures that are known to prevent and deal with any deviations from the rules of ethics. This commitment involved the appointment of research integrity officers (RIOs). The “*Office Français de l’Intégrité Scientifique*” (OFIS) was installed in March 2017. The OFIS has three missions: platform for reflection (it contributes to the definition of a national policy on scientific integrity); observation (it leads a national observatory on the implementation of the commitments of the charter); and animation (it leads and promotes the work of the network of RIOs of the institutions). The OFIS does not manage claims from whistle-blowers and has no power to investigate cases of malpractices.

Parallel to the QRPs description, a crisis of reproducibility has been observed that is partially related to malpractices [2]. Early-career researchers are concerned with the costs of reproducibility and have difficulty maintaining a boundary between responsible research conduct and QRPs when they observe the practices of senior researchers. “First, we must acknowledge that we have a problem, and that all of our careers have benefitted from the research practices that we now realize to be *questionable*,” RA Poldrack stated [3]. Following this question from early-career researchers, a new question arises: are senior researchers the right people to manage QRPs?

Since 2015, there has been no assessment of the progress of research integrity (RI) and RIOs nominations in France. There is no annual report published as yet on cases of misconduct, either at the level of each RIU or by the OFIS.

Our objectives were to search for information to describe the RI development and to analyze the RIOs’ profiles. We used the information available from OFIS and public websites.

## Methods

The OFIS website (https://www.hceres.fr/fr/office-francais-de-lintegrite-scientifique) lists all public RIUs and their designated RIOs. The French RIUs are classified (OFIS website) into four groups: (1) universities, which deliver doctoral degrees; (2) schools (“écoles”), including public schools for engineers, specialists in social sciences, etc.; (3) research agencies (“instituts de recherche”), including national research institutions and funders; (4) and national higher education schools (called “grands établissements”) that train students in general fields. The names of the RIOs are listed on the OFIS website for each RIU that discloses the information to the OFIS. The private schools, which mostly include business and management schools (delivering diplomas such as Masters in Business Administration), are not listed on the OFIS website.

During the period between 6 and 14 October 2018, and on 2 May 2019 (in order to update our data before the June World Conference on Research Integrity), we entered two keywords (“*plagiat*’ and ‘*intégrité*”) into the search engines on the RIU homepages and retrieved the relevant information (including “research” and “scientific” integrity when “intégrité” was entered). We consulted the governance and downloaded the organigram to determine whether the RIO positions and names were mentioned. We believe that integrity and plagiarism are the most common terms used by students and researchers who are not familiar with verbiage related to integrity (for example, QRPs, RIOs, reproducibility) and who are looking for information on a site. We have chosen these terms for the search on the websites.

We searched the domains for expertise, sex, and age of the RIOs from their curriculum vitae (institutional websites), LinkedIn profiles, and various Google links. The domains were classified as follows: “engineer,” including engineering/computer science/mathematics; “social sciences and humanities” (SSH), including social sciences/philosophy/history; “biology,” including biological sciences/medicine/veterinary/agronomy; physics/chemistry; “law,” including politic/economy; and others.

We determined the age of the RIOs from the resumes available on the Internet (RIUs site and/or social networks). When age was not retrieved (for researchers over 70 years), we found the PhD thesis date, and assumed that it was passed when they were 27 years old (sciences), 30 (law, economy), or 34 (social sciences), according to French data [4]. We estimated their age based on the date of obtaining the PhD thesis and since we had their age at the date of their PhD, we extrapolated their age to 2019. We considered 65 years as the retirement age in the public system, and that the RIOs who were below this age were active.

## Results

The OFIS website lists 142 RIUs (Table 1). Searching for the keyword “plagiarism” retrieved 25 RIUs; however, the web information was minimal, and consisted entirely of charters, interviews, and rare training modules. The keyword “integrity” turned up 23 RIUs. Nonetheless, there was little information available beyond notices for seminars, events, and a few training modules.

**Table 1:** Information retrieved from 142 French research institutions and universities regarding their research integrity practices, with characteristics of research information officers (RIOs)

	Total	Universities	“Schools”^a^	Research agencies	National higher education schools ^a^
Number	142	79	28	21	14
Websites					
Plagiarism	25	19	3	2	1
Integrity	23	12	3	7	1
RIOs on organigram	13	8	0	4	1
RIOs characteristics					
RIOs number (%)	96 (67.6)	54 (68.4)	13 (46.4)	19 (90.5)	10 (71.4)
M/F	68/28	39/15	11/2	11/8	7/3
Age (mean + SD)^b^	61.2 + 9.6	61.4 + 10.4	60.3 + 5.4	61.0 + 11.3	61.8 + 7.2
> 65 years	38	25	3	6	4
< 50 years	14	11	0	3	0
51 to 64 years	44	18	10	10	6
RIOs domains of expertise^c^					
Engineer	25	13	4	7	1
SSH^d^	22	15	2	3	2
Biology	22	10	2	7	3
Physics/chemistry	13	5	4	1	3
Law	11	9	0	1	1
Others	3	2^e^	1^f^	0	0

^a^See methods for definition of “schools”

^b^6 missing data; 3 for universities, 1 for schools, 2 for research agencies

^c^See methods for definition of the domains

^d^*SSH* Social Sciences and Humanities

^e^Architect and archeology

^f^Librarian

Of the total 142 RIUs, 66.2% (*n* = 94) had named 96 RIOs (two of these had two RIO positions: one for science/technology/medicine, and one for humanities and social sciences). Furthermore, 29.2% RIOs (*n* = 28) were female and 70.8% (*n* = 68) were male. Out of the 96 RIOs, 58 were active (< 65 years old) and had an RIO function added to their usual laboratory activities. Two of the 58 active RIOs occupied a full-time RIO position. The mean age of 90 RIOs (6 missing data) was 61.2 0+ 9.6 years (range 35–76). The breakdown by domain of expertise and type of RIU is shown in the Table 1.

## Discussion

There is a lack of information about RI on the websites of French universities and research organizations, which may reflect a lack of information and commitment in the institutions themselves. We have demonstrated that few RIUs among the 142 provided information about plagiarism/integrity on their websites, and only 13 RIOs out of 96 were clearly named on the RIU website. Nearly 40% (38/96) of the RIOs were older than 64 years and had probably retired. The fact that RIOs are retired can be an advantage for the institution because they have time for their mission and do not have any agenda that conflicts with their priorities. However, there are disadvantages as well because they tend to protect the institution to avoid a bad image, and they lack experience in new laboratory practices. This observation requires further research.

Our observations can be compared to other countries. For example, the *Concordat to Support Research Integrity* published in 2012 recommended that UK research institutions should provide a named point of contact to address concerns about RI. Over 6 years after the publication of the *Concordat*, nearly half of the UK universities were not compliant with all its recommendations and provided neither the contact details for staff members responsible for RI nor annual statements [5]. As in the UK, we have observed a variability in the profiles of the RIOs, and most RIOs in professional activity do not have time clearly dedicated to their activity. On the other hand, retired RIOs seem to have a lot of time for their mission. Is the UK system better performing than the French one? The answer depends on how both countries are compared. Implementing RI in all countries is time-consuming, which can be considered normal when practices require changes. Nearly 4 years after the national charter was signed, it is obvious that the development of RI in France has been slow.

France does not have an institution similar to the US Office of Research Integrity (ORI), which investigates falsification, fabrication, and plagiarism, apart from making decisions. In France, it is not possible for the OFIS to investigate allegations of misconduct. Rather, the OFIS is expected to coordinate the policies for RI and to observe the practices of researchers and institutions. The USA does not have an organization equivalent to the OFIS. A report of the National Academies of Sciences, Engineering, and Medicine has called (recommendation 4) for the creation of a Research Integrity Advisory Board that would play a similar role to the OFIS [6].

We should question the competencies of the RIOs as there is no national “job description” provided. RIOs have been locally named, with institutions having only two alternatives: either an active researcher with little time is dedicated to the additional RIO responsibility or a retired researcher who can devote time to the mission is appointed RIO, despite being distanced from laboratory practices. Most RIOs are full-time researchers themselves and are, therefore, ill-equipped to assume additional responsibilities such as promoting ethical conduct and practices. There is no description of all the competencies required to carry out the mission of an RIO, but the national network of RIOs aims to harmonize practices. This network, which is periodically convened, is a good initiative to set up a system of scientific integrity. Over a period of time, it will be possible to better define the profile and skills required of RIOs (for example, completing scientific integrity training). The retired RIOs can protect the institution and prioritize their opinions over the facts; however, this is yet to be demonstrated. Because the practice of scientific research in 2019 is not the same as the science of the 1980s, early-career researchers could not easily accept a senior RIO investigating practices that he or she had never experimented with.

It would be useful to have a specification for RIUs to encourage them to publish information on QRPs, especially regarding their policies. When a researcher wants to report malicious intent, he or she has great difficulty in identifying the person who can act upon his or her request.

## Conclusion

There is a lack of information about RI on the websites of French universities and research organizations, which may reflect a lack of information and commitment in the institutions themselves. The system for RI is still under development, and not all its rules can be implemented simultaneously. Therefore, more time is necessary to successfully implement all its rules. Interviews with RIOs who have diverse experiences will be useful in defining the expected competencies as well as the needs of the RIUs.

## Data Availability

The datasets used and/or analyzed in this study are available from the corresponding author on reasonable request.

## Abbreviations

OFIS: Office Français de l’Intégrité Scientifique
QRPs: Questionable research practices
RI: Research integrity
RIO: Research integrity officers
RIU: Research institutions and universities
SSH: Social sciences and humanities
